# Tailoring Polymeric Binder of Permselective Gas Diffusion Electrode for Low‐Concentration CO_2_ Electrolysis

**DOI:** 10.1002/cssc.202501145

**Published:** 2025-10-21

**Authors:** Hadi Shaker Shiran, Shariful Kibria Nabil, Tareq Al‐Attas, Karthick Kannimuthu, Soumyabrata Roy, Md Golam Kibria

**Affiliations:** ^1^ Department of Chemical and Petroleum Engineering University of Calgary 2500 University Drive, NW Calgary Alberta T2N 1N4 Canada; ^2^ Department of Sustainable Energy Engineering Indian Institute of Technology Kanpur Kanpur Uttar Pradesh 208016 India

**Keywords:** electrolysis, lean CO_2_, metal‐organic‐framework, permselective, polymer

## Abstract

Electrochemical CO_2_ reduction (eCO_2_R) offers a promising route to convert industrial CO_2_ emissions into value‐added chemicals. However, direct electrolysis of low‐concentration CO_2_ streams from flue gas suffers from mass transport limitation, resulting in poor Faradaic efficiency (FE). To address this challenge, a gas diffusion electrode featuring a permselective layer is developed that selectively concentrates CO_2_ at the catalyst interface. The permselective layer integrates a hybrid CO_2_‐philic metal‐organic framework Calgary Framework‐20 (CALF‐20) filler embedded within a tailored polymer matrix. Three polymers—Nafion, polysulfone (PSF), and styrene‐ethylene‐butylene‐styrene—as the polymer matrix to optimize CO_2_/N_2_ selectivity and permeability are systematically tested. The CALF‐20/PSF composite in the permselective layer achieves a CO_2_/N_2_ selectivity of ≈40, enabling a threefold increase in CO partial current density (*j*_CO_ of −42.7 mA cm^−2^) with a dilute CO_2_ feed (10 volume percentage) compared to unmodified electrodes at an applied current density of −50 mA cm^−2^. In a membrane electrode assembly, the optimized electrode maintains a stable FE_CO_ of ≈70% for over 20 h. CO_2_ uptake studies and structural characterization reveal that strong interactions between the triazole ligands of CALF‐20 and the sulfonyl/ether groups of PSF enhance both CO_2_ transport and electrode durability.

## Graphical abstract

Selective CO_2_ permeation through a metal‐organic framework–polymer membrane boosts CO partial current density in lean flue gas electrolysis.

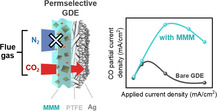

## 1 Introduction

The unrestrained global combustion of fossil fuels has noticeably increased the atmospheric concentration of carbon dioxide (CO_2_) to alarming levels. Integrated CO_2_ capture and conversion has emerged as a promising strategy to mitigate anthropogenic CO_2_ emissions.^[^ ^1^, ^2^ ^]^ Electrochemical CO_2_ reduction (eCO_2_R) offers multiple benefits, including scalability, ambient operation conditions, and stackable configuration, enabling efficient conversion of CO_2_ into value‐added products. When powered by renewable electricity and integrated with conventional CO_2_ capture processes, eCO_2_R can serve as a forefront technology in the transition to a net‐zero future.^[^ ^2^, ^3^ ^]^

**Figure 1:**
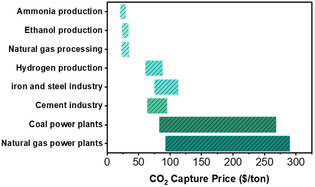
Estimated range of CO_2_ capture cost from different point sources in the United States.^[^ ^4^ ^]^

Despite advancements, large‐scale deployment of CO_2_R remains economically challenging due to high carbon capture costs, which vary significantly with CO_2_ concentration.^[^ ^4^ ^]^ For instance, CO_2_ capture costs from ammonia production, ethanol production, and natural gas processing plants are relatively low (≈$50 per ton) as the concentration of CO_2_ in the flue gas from these industries is relatively high (>30 vol%)^[^ ^5^ ^]^ (**Figure** **1**). Conversely, ≈73% of industrial CO_2_ emissions originate from power generation point sources, where CO_2_ concentrations are low (≈10 vol%) (Figure S1, Supporting Information). This leads to increased capture costs (up to $270 per ton CO_2_) (Figure 1).^[^ ^5^ ^]^

Recent efforts have explored the direct electrolysis of low‐concentration CO_2_ feeds to circumvent high capture costs.^[^ ^6^, ^7^, ^8^, ^9^, ^10^ ^]^ Tan et al. demonstrated that the CO_2_ concentration in the feed stream significantly affects the local CO_2_ concentration in the reaction interface, thereby influencing the long‐term performance of eCO_2_R systems.^[^ ^11^ ^]^ Thus, developing cost‐effective carbon capture and conversion technologies is imperative to address the CO_2_ feedstock constraint and make the overall end‐to‐end process economically feasible.

An alternative integrated approach involves the direct electrochemical conversion of CO_2_‐rich amine solutions (e.g., monoethanolamine,^[^ ^12^ ^]^ ethylenediamine, and diethanolamine^[^ ^13^ ^]^). However, challenges such as mass transfer limitations, poor catalyst stability, and the toxic nature of amine solutions hinder their large‐scale adoption.^[^ ^14^, ^15^ ^]^ Similarly, aqueous (bi)carbonate electrolysis has been reported to bypass the energy‐intensive solvent regeneration and CO_2_ compression stages of incumbent capture systems.^[^ ^16^, ^17^, ^18^ ^]^ Nonetheless, this approach faces limitations, including low partial current density due to mass transport challenges, limited mechanistic understanding, and low energy efficiency, which hinder its overall practicability.^[^ ^14^, ^19^ ^]^

Motivated by the economic feasibility of the direct conversion of flue gas (avoiding regeneration, compression, and transportation steps), we have recently proposed a permselective gas diffusion electrode (PGDE) for integrated eCO_2_R.^[^ ^20^ ^]^ We fabricated the PGDE with a permselective mixed matrix membrane to selectively permeate CO_2_ from a diluted gas stream. Among the various inorganic fillers for the membrane, metal‐organic frameworks (MOFs) are of particular interest owing to their unique advantages, including high surface area, scalability, and tunable pore properties.^[^ ^21^ ^]^ We demonstrated that Zn‐based MOF Calgary Framework‐20 (CALF‐20) as filler within a polymer matrix (i.e., Nafion) offers high separation efficiency for CO_2_ in point source flue gas (additional details presented in Methods in the Supporting Information). Our detailed analysis revealed that the CO_2_‐philic nature of the membrane maintains CO_2_ partial pressure and thus increases the dissolved CO_2_ concentration, leading to higher CO_2_ availability within the catalyst vicinity for eCO_2_R. Cite that paper again

CALF‐20 is known for its remarkable CO_2_ selectivity and sorption attributes, as demonstrated in previous works.^[^ ^22^, ^23^ ^]^ However, a key limitation was the relatively low partial current density compared to pure CO_2_‐fed eCO_2_R. This performance discrepancy originated from the mass transport limitation of CO_2_ at elevated current densities. Due to the inherent selectivity/permeability trade‐off in mixed matrix membrane—where a highly permeable membrane lacks selectivity and vise versa^[^ ^24^ ^]^—structural modifications are necessary to achieve an optimal balance. While CALF‐20 ensures high permeability without compromising selectivity, the polymer matrix plays a crucial role in further enhancing CO_2_/N_2_ selectivity for efficient CO_2_ transport within the PGDE.^[^ ^25^ ^]^

We hypothesize that optimizing the polymer matrix can mitigate these trade‐offs by selectively facilitating the transport of sorbed CO_2_ to the catalyst interface. This approach would enable a synergistic effect, where the high selectivity of MOFs and improved mass transport facilitated by the polymer matrix collectively enhance electrochemical performance.

Herein, we systematically investigate different polymer matrices—Nafion, polysulfone (PSF), and styrene‐ethylene‐butylene‐styrene (SEBS)—in permselective membranes embedded with MOF/polymer layers (MOFPLs). The study aims to develop a PGDE optimized for low‐concentration CO_2_ electrolysis. Additionally, we compare CALF‐20 with the commercially available zeolitic imidazolate framework‐8 (ZIF‐8) in MOFPL‐derived PGDEs. ZIF‐8, comprising Zn^2+^ nodes linked by 2‐methylimidazole ligands in zeolitic sodalite (SOD) topology,^[^ ^26^, ^27^ ^]^ is widely studied as a filler in MOFPLs. Our results show that the CALF‐20/PSF composite of MOFPL exhibits a threefold increase in partial current density for CO production compared to ZIF‐8/PSF and the bare (unmodified) GDE. The CALF‐20/PSF‐based PGDE can maintain ≈70% FE_CO_ over 20 h at 30 mA cm^−2^, demonstrating its potential for efficient eCO_2_R from lean CO_2_ feedstocks.

## 2 Results and Discussion

We initiated our study by evaluating an unmodified sputtered silver (Ag) catalyst on polytetrafluorethylene (PTFE) based GDE to investigate the effects of varying CO_2_ partial pressure on eCO_2_R (experimental details are available in Methods). We performed the experiments with 10, 30, 50, 70, and 100 vol% CO_2_ concentrations (and N_2_ balance) and analyzed the partial current densities (Figure S2, Supporting Information). Under pure CO_2_, we could achieve CO partial current density (*j*_CO_) exceeding −95 mA cm^−2^ at −100 mA cm^−2^ applied current density (*I*_applied_). In contrast, diluted CO_2_ feedstocks significantly reduced performance, with *j*_CO_ dropping to a maximum of −49 mA cm^−2^ at *I*_applied_ of 100 mA cm^−2^ for 70% CO_2_. These results dictate that reduced CO_2_ concentrations lead to a sharp decline in *j*_CO_, likely due to limited CO_2_ availability in the catalyst vicinity.^[^ ^11^ ^]^

To address this limitation, we engineered GDEs modified with permselective membranes embedded with MOFPLs to enhance CO_2_ mass transport at the reaction interface.^[^ ^20^ ^]^ To overcome the selectivity/permeability trade‐off, we screened polysulfone (PSF) and SEBS alongside Nafion as polymer matrices for PGDEs. PSF and SEBS exhibit robust thermal and chemical stability, tunable mechanical properties, and a wide range of CO_2_/N_2_ selectivity and CO_2_ permeability (13–39.5 and 17.9–170 Barrers, respectively) as summarized in Table S1, Supporting Information. Fabrication parameters for the PGDEs (mass loading and ratio, deposition technique, drying sequence, etc.) are detailed in SI.

**Figure 2:**
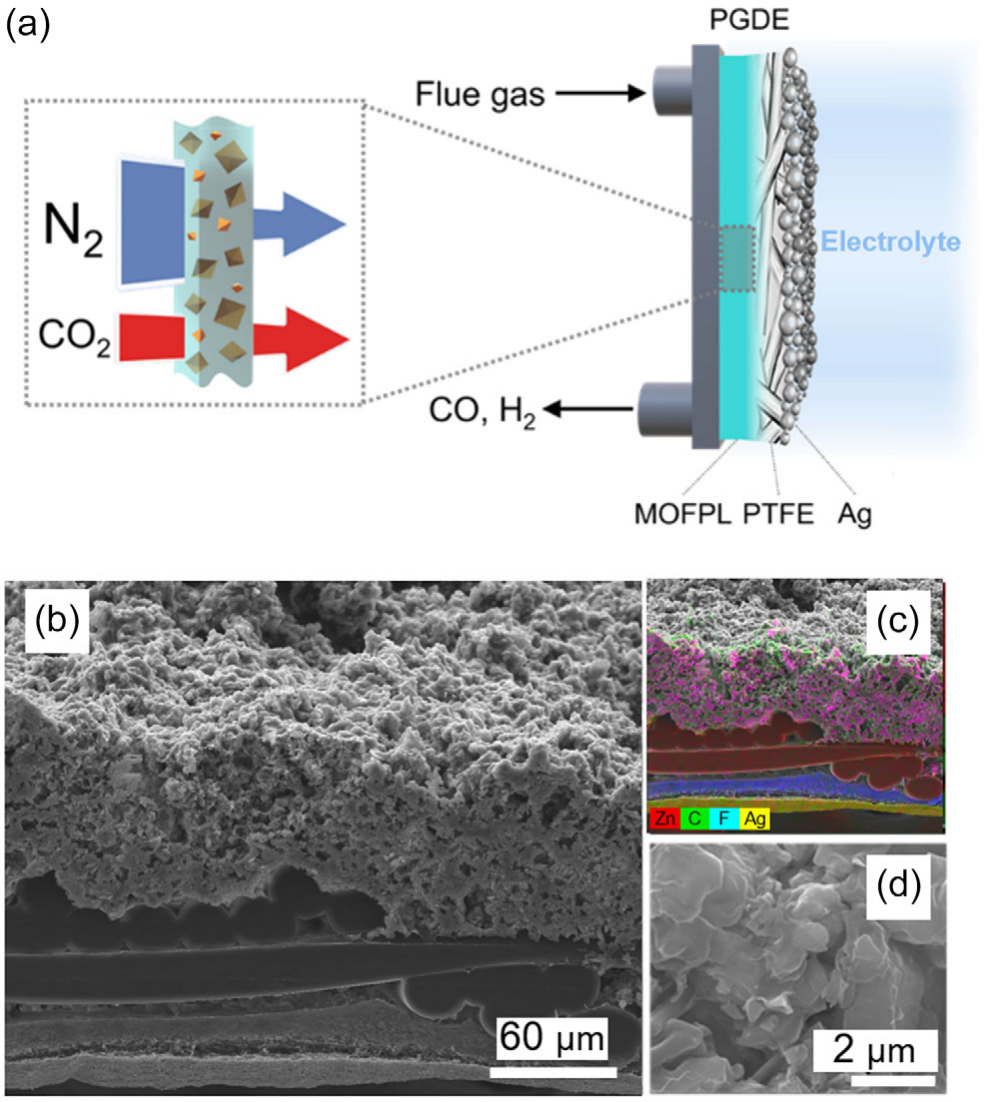
Demonstration of the PGDE hypothesis. a) Schematic presentation of the MOFPL at the gas diffusion side of PGDE, b–d) SEM images of the PGDE.

**Figure** **2**a illustrates the PGDE design for eCO_2_R using diluted CO_2_ streams, indicating MOFPL placement at the rear of the Ag/PTFE electrode. As shown in the magnified view, MOFPL allows selective permeation near the catalyst surface, improving eCO_2_R efficiency.^[^ ^20^, ^28^ ^]^ Cross‐sectional scanning electron microscopy (SEM) confirmed uniform MOFPL integration with the Ag/PTFE (Figure 2b–d). Energy dispersive X‐ray of the CALF‐20/PSF composite (Figure S3, Supporting Information) verified the presence of key elements: carbon and fluorine (PTFE backbone), sulfur (PSF polymer), zinc (CALF‐20), and silver (sputtered Ag catalyst).

**Figure 3:**
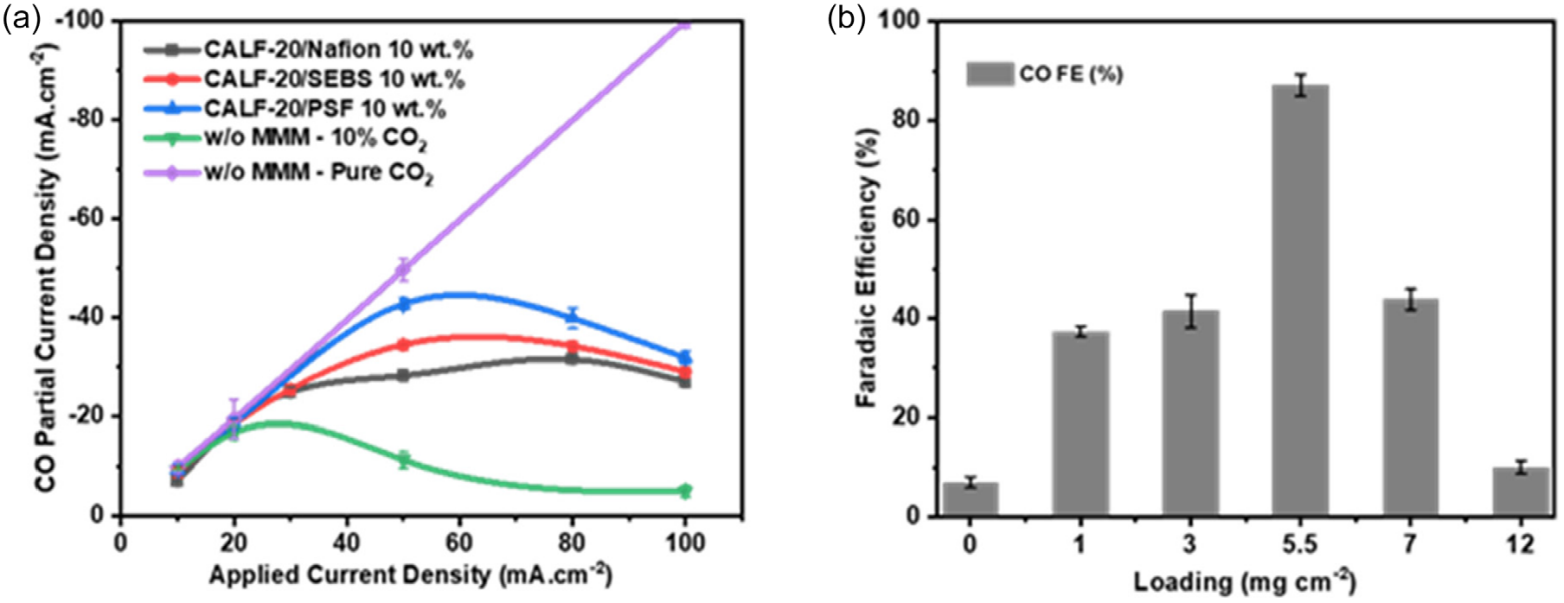
a) Partial current density of CO_2_ reduction to CO using GDEs modified with CALF‐20‐based MOFPL, incorporating Nafion, SEBS, and PSF ionomers, compared to the bare GDE. The experiments include a 10 vol% CO_2_ feed stream, alongside a control experiment with the bare GDE under a pure CO_2_ feed stream. The 10 wt% refers to the weight percentage of polymer in the MOF‐polymer composite layer, i.e., 10 wt% polymer and 90 wt% MOF. b) Effect of CALF‐20/PSF loading on CO FE in an AEM‐based membrane electrode assembly.

Subsequently, the performance of PGDEs with the different polymer matrices were evaluated under a diluted CO_2_ stream (10 vol% and balance N_2_). As shown in **Figure** **3**a, MOFPLs with all polymers incorporating CALF‐20 as the MOF filler exhibited substantially higher *j*_CO_ compared to the bare GDE. Among these, the PSF‐based PGDE achieved the highest partial current density for CO at −42.7 mA cm^−2^ under an *I*_applied_ of 50 mA cm^−2^, outperforming SEBS (−34.5 mA cm^−2^) and Nafion (−31.5 mA cm^−2^) counterparts. However, for all cases (except pure CO_2_), *j*_CO_ decreases at higher total current densities. In general, *j*_CO_ and CO_2_ partial pressure (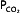) reveal a near‐linear relationship under low‐to‐moderate current densities (Figure S2, Supporting Information), indicating that CO_2_ availability is a key rate‐limiting factor for the eCO_2_R. However, at low 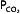 (10 vol%) and elevated applied currents, *j*_CO_ begins to decline due to severe mass transport limitations as well as undesired side reactions (i.e., (bi)carbonate formation). These conditions limit the flux of CO_2_ near the catalyst interface, leading to surface depletion and reduced reaction rates. This transition from kinetically controlled to diffusion‐limited behavior at low 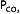 has also been reported in prior studies.^[^ ^29^, ^30^ ^]^ Hence, the introduction of the MOF‐based permselective layer mitigates this effect by selectively enriching CO_2_ over N_2_ near the catalyst surface, improving *j*_CO_ retention under low 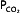 operation.

To rationalize these results, we compared the CO_2_/N_2_ selectivity and permeability of the polymers metrices. Figure S4, Supporting Information details the reported CO_2_/N_2_ selectivity ranges and CO_2_ permeability of pure Nafion, SEBS, and PSF membranes relative to Robeson's upper bound, which defines the empirical trade‐off limit between permeability and selectivity for a given pair of gases like CO_2_/N_2_ (Table S1 and S2, Supporting Information). PSF demonstrates the highest CO_2_/N_2_ selectivity while maintaining permeability near Robeson's upper bound, followed by SEBS and Nafion. The observed eCO_2_R of the respective PGDEs directly correlates with these polymer properties, supporting the hypothesis that enhanced CO_2_/N_2_ selectivity and permeability improve *j*_CO_ by increasing CO_2_ availability at the reaction interface.

To assess the impact of MOFPL loading on electrochemical performance, electrochemical CO_2_ reduction experiments were conducted across different loadings of CALF‐20/PSF composite ranging from 0 to 12 mg cm^−2^ (Figure 3b). Increasing the loading from 0 to 5.5 mg cm^−2^ significantly improved the FE_CO_. However, at loadings >5.5 mg cm^−2^, the FE_CO_ faradaic efficiency (FE) started to decrease, suggesting that the higher MOFPL thickness blocks the diffusive pores of GDE and limits the CO_2_ diffusion to the catalyst surface.^[^ ^28^ ^]^

**Figure 4:**
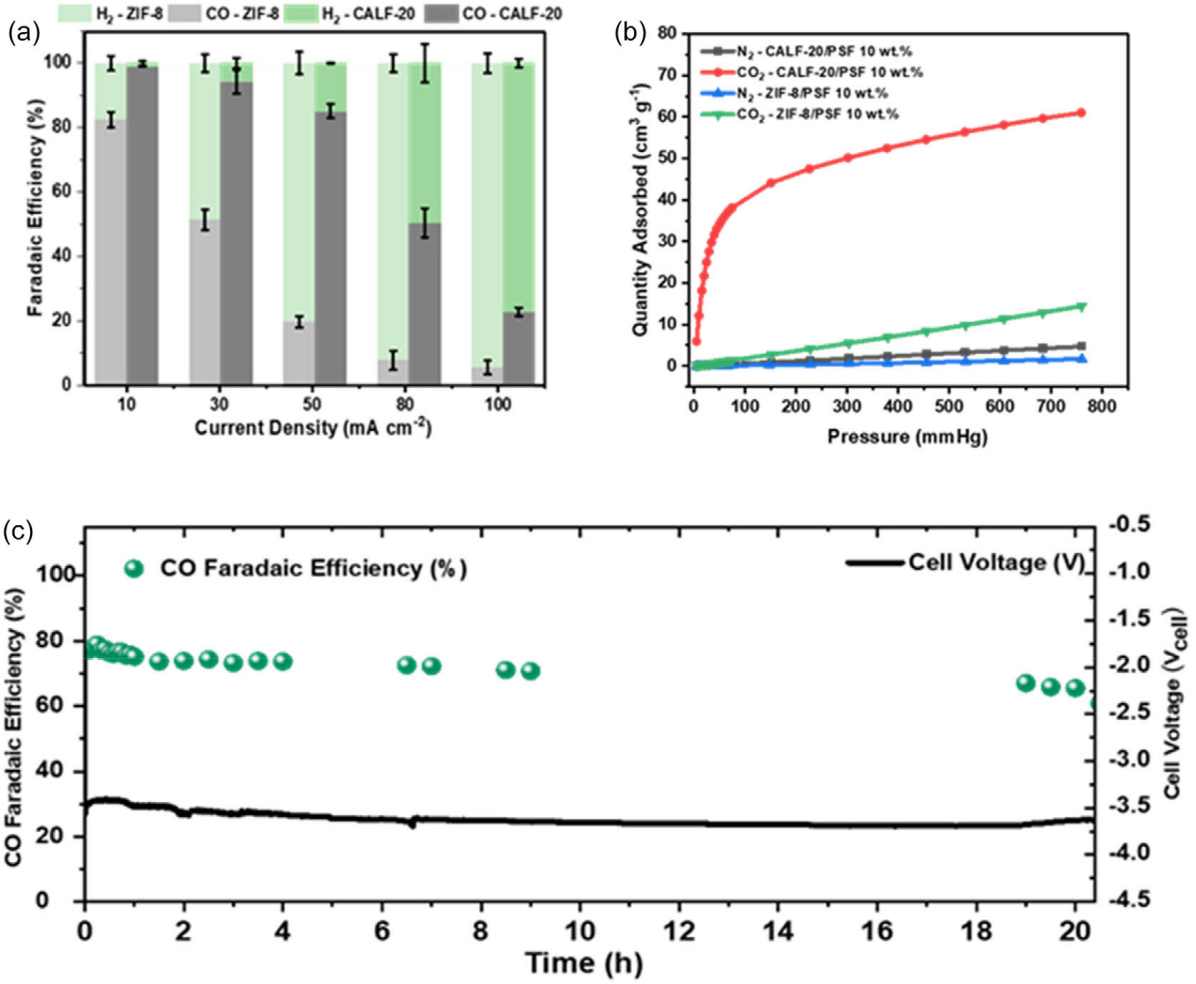
a) FE_CO_ of CALF‐20/PSF versus ZIF‐8/PSF at different *I*_applied_ using an AEM‐based MEA, b) Adsorption isotherms of CALF‐20/PSF and ZIF‐8/PSF, and c) Extended run of CALF‐20/PSF at 30 mA cm^−2^ under eCO_2_R environment (using a BPM‐based MEA equipped with Ni foam anode and 1 M KOH anolyte). All experiments were performed with 10 vol% CO_2_ and balance N_2_.

While MOFs are primarily incorporated into the MOFPL layer to enhance permeability, they also improve membrane selectivity. To investigate this dual role, we compared CALF‐20 and ZIF‐8 embedded in PSF‐based MOFPL. As shown in **Figure** **4**a, the PGDE with CALF‐20/PSF MOFPL consistently outperformed the ZIF‐8/PSF counterpart across all applied current densities, achieving higher FE for CO, indicative of superior permselectivity.

Gas adsorption isotherms (Figure 4b) revealed that CALF‐20/PSF exhibits four orders of magnitude higher CO_2_ uptake capacity than ZIF‐8/PSF, corroborating its enhanced FE_CO_. CALF‐20 maintains CO_2_‐philicity and structural stability at high relative humidity (RH) (>80% RH).^[^ ^23^, ^31^ ^]^ In contrast, ZIF‐8 undergoes hydrolysis at >50% RH, decomposing into zinc and imidazolate ions.^[^ ^32^, ^33^ ^]^ Fourier‐transform infrared spectroscopy (FTIR) of ZIF‐8/PSF PGDEs pre‐ and post‐reaction (2 h) confirmed water absorption by the membrane, evidenced by a broad O—H stretching peak at ≈3380 cm^−1^ (Figure S5, Supporting Information). Although dry CO_2_ was supplied, water diffusion from the anode via the anion exchange membrane elevated cathode RH to 79%,^[^ ^34^ ^]^ likely destabilizing ZIF‐8 and reducing CO_2_ availability. In contrast, CALF‐20 retained structural integrity under high RH, as verified by FTIR (Figure S6, Supporting Information).

While anion exchange membranes (AEMs) facilitate efficient ion transport at moderate applied currents,^[^ ^35^, ^36^ ^]^ the higher current densities achieved by our PGDEs introduced new challenges. Specifically, CO_2_ loss via (bi)carbonate formation and crossover to the anode lowers CO_2_ utilization and leads to cathode salt accumulation, causing flooding and compromising system stability.^[^ ^35^, ^37^, ^38^ ^]^ To address these limitations, we employed a bipolar membrane (BPM), which suppresses (bi)carbonate formation and crossover (Figure S7, Supporting Information).^[^ ^37^, ^39^ ^]^ Long‐term stability testing with BPM demonstrated sustained operation at an applied current density of 30 mA cm^−2^ for 20 h, maintaining an FE_CO_ of 70 ± 5% at a full‐cell voltage of ≈−3.50 V_cell_ (Figure 4c).

**Figure 5:**
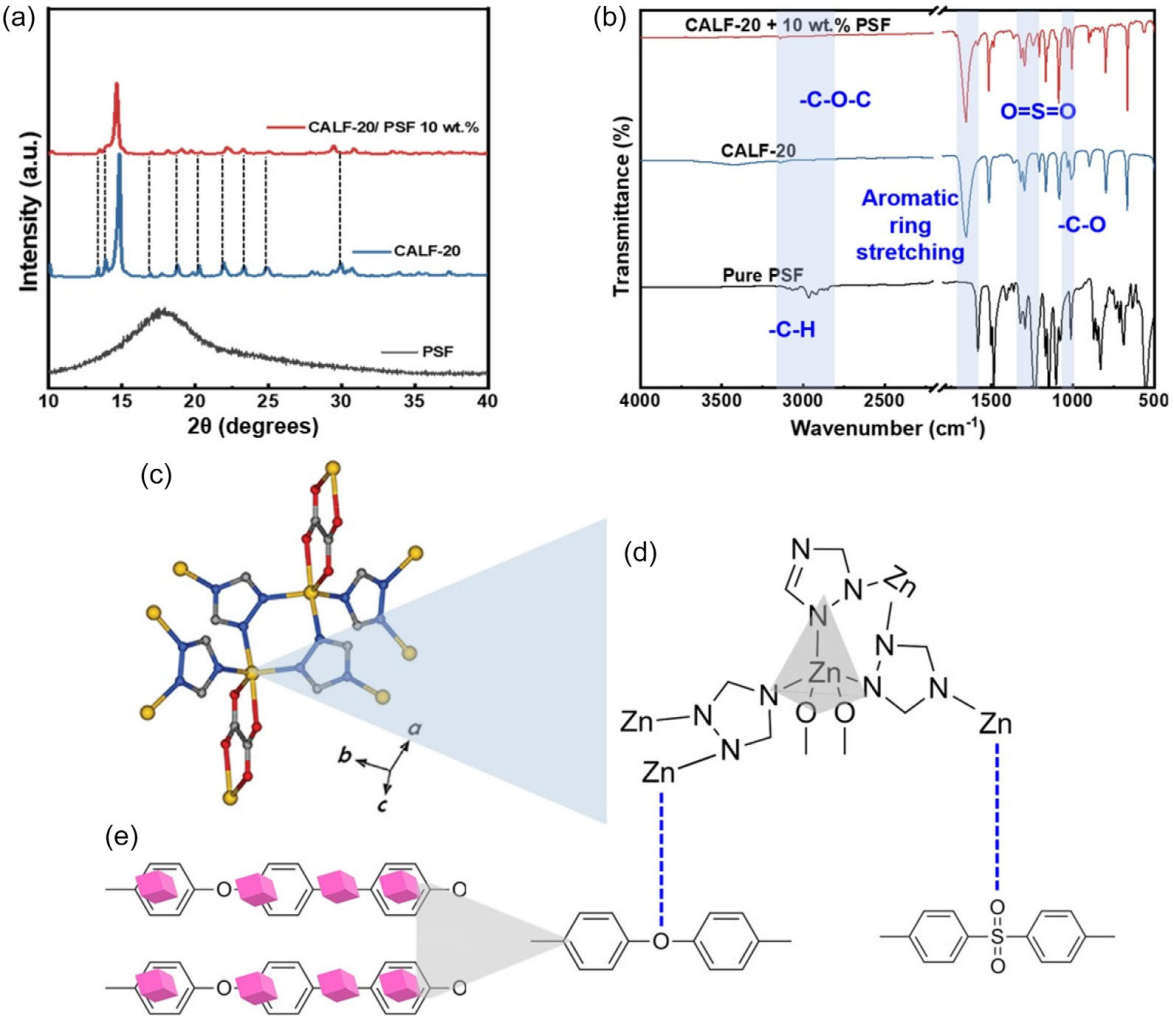
Characteristic interactions between CALF‐20 and PSF. a) XR pattern of pristine CALF‐20, PSF, and PGDE (CALF‐20/PSF), b) FTIR spectral studies of CALF‐20/PSF, and c–e) CALF‐20/PSF model structure showing the interactions of Zn with the polymer (PSF). The pink cubes in (e) represent CALF‐20 to demonstrate its interactions with PSF.

The MOF‐polymer interactions in the permselective layer (MOFPL) govern structural stability, thermal robustness, and gas transport efficiency, directly enhancing and sustaining eCO_2_R performance. X‐ray diffraction (XRD) analysis (**Figure** **5**a) revealed structural distortions in CALF‐20/PSF, evidenced by reduced peak intensities and a main peak left shift (14.8° → 14.6°), attributed to hydrogen bonding between PSF sulfone groups and CALF‐20 triazole ligands, indicating well dispersion of particles. Density functional theory studies confirm triazole protons form H‐bonds with CO_2_ oxygens, enhancing adsorption.^[^ ^23^ ^]^ Moreover, Zn‐coordinated triazole units (via N‐lone pairs) leave adjacent carbons electron‐deficient,^[^ ^40^ ^]^ enabling ionic interactions with PSF sulfone groups (≈15% ionic character).^[^ ^23^ ^]^

Vibrational spectroscopies further elucidated these interactions. FTIR (Figure 5b) confirmed coexisting PSF (sulfonyl, ether) and CALF‐20 (triazole, oxalate) functional groups,^[^ ^41^, ^42^, ^43^ ^]^ with band shifts indicating Zn‐PSF coordination.^[^ ^44^, ^45^, ^46^ ^]^ PSF exhibits characteristic FTIR bands at 2800–3000 cm^−1^ (C—H and C—O—C—CH_3_ stretching), ≈1590 cm^−1^ (aromatic ring), 1155 and 1295 cm^−1^ (O=S=O stretches), 1320 and 1015 cm^−1^ (C—SO_2_—C and C—O stretches), and ≈1170 cm^−1^ (etheric C—O—C).^[^ ^41^ ^]^ Pristine CALF‐20 shows bands at ≈3100 cm^−1^ (C—H from triazole), 1560 cm^−1^ (C=N), 1660 cm^−1^ (C=O), 1350–1550 cm^−1^ (ring strain), along with low‐frequency bending vibrations, consistent with its Zn–triazolate–oxalate framework.^[^ ^42^, ^43^ ^]^ In CALF‐20/PSF (10 wt%), the spectrum is dominated by CALF‐20 bands, but weak PSF ether‐linkage bands at 2800–3000 cm^−1^ indicate possible Zn^2+^—O interactions.^[^ ^23^ ^]^ Raman spectra (Figure S8, Supporting Information) also confirmed Zn‐PSF coordination with reduced intensity of PSF peaks at 790 and 1140 cm^−1^, suggesting restricted polymer mobility due to interactions with CALF‐20.^[^ ^41^, ^47^ ^]^ Figure 5c–e illustrates the interaction mechanism between CALF‐20 and PSF, where Zn sites in the CALF‐20 coordinate with ether and sulfone oxygen atoms from PSF. This Zn—O coordination promotes interfacial compatibility and suggests favorable polymer alignment at the MOF surface for enhanced performance.^[^ ^22^ ^]^ Lastly, thermogravimetric analysis (Figure S9, Supporting Information) demonstrated lower weight loss for the CALF‐20/PSF composite compared to pristine CALF‐20 at 350 °C, confirming strong interfacial interactions.^[^ ^48^ ^]^ Further details on the structural changes upon forming the MOFPL composite are available in Supplementary Note 1, Supporting Information.

## 3 Conclusions

This study demonstrates that tailoring the polymer matrix in MOFPL‐based PGDEs significantly enhances electrochemical CO_2_ reduction performance for lean CO_2_ feedstocks. The MOFPL with CALF‐20/PSF emerged as the optimal configuration, achieving a CO partial current density of −42.7 mA cm^−2^ at 50 mA cm^−2^ applied current density, outperforming both ZIF‐8/PSF and unmodified electrodes. With a CO_2_ uptake capacity >4 orders of magnitude higher than ZIF‐8/PSF, the CALF‐20/PSF PGDE delivered a 65% improvement in FE_CO_ and sustained stable operation for over 20 h. Structural characterization via FTIR, Raman spectroscopy, and XRD revealed robust interactions between CALF‐20 and PSF, mediated by sulfonyl/ether linkages and electron‐deficient triazole sites, which enhanced mechanical stability and CO_2_ transport efficiency. These findings underscore the critical role of polymer‐MOF synergy in balancing selectivity and permeability for efficient eCO_2_R. Future work should prioritize rational design of MOF‐polymer interfaces through advanced synthesis techniques, computational modeling, and operational optimization (e.g., temperature/pressure modulation). Direct measurement of CO_2_ permeation/effluent concentration through each MOFPL would provide further insight. Still, the observed electrochemical performance trends align well with reported CO_2_/N_2_ selectivity values for the respective polymers (Table S1, Supporting Information). Finally, evaluating PGDE performance in realistic flue gas environments containing O_2_, moisture, and acidic gases will be essential for scaling this technology.

## 4 Experimental Section

4.1

4.1.1

##### CALF‐20 Synthesis

CALF‐20 was synthesized using the procedure reported elsewhere1. Briefly, a mixture of zinc oxalate dihydrate and 1,2,4‐triazole (1:1 mole ratio) was prepared in 100% pure ethanol. The mixture resulted in a milky white and opaque solution, which was poured into a Teflon liner and sealed in a stainless‐steel autoclave. The autoclave was then heated to 180 °C for 48 h in an oven. The resulting material was filtered and washed with ethanol.

##### Preparation of Ag/PTFE Gas Diffusion Electrode (GDE)

A 300 nm thick layer of silver was sputtered on a polytetrafluoroethylene (PTFE) substrate using a Kurt J. Lesker CMS‐18 system. The sputtering rate was 40 nm min^−1^ at 300 W, and argon was used as the carrier gas with a flow rate of 139 cm^3^_STP_ min^−1^ (sccm).

##### Preparation of the Permselective Gas Diffusion Electrodes (PGDE)

MOFPL solutions were prepared with a composition of 10 wt% polymer and 90 wt% MOF. The polymers used were SEBS (Polystyrene‐block‐poly(ethylene‐ran‐butylene)‐block‐polystyrene, with an average molecular weight of 89 000 by Gel Permeation Chromatography (GPC) and was in the form of coarse powder obtained from Sigma‐Aldrich) and PSF (Polysulfone, with an average molecular weight of 35 000 by light scattering and average number‐average molecular weight of 16 000 by melt osmometry (MO), in the form of transparent pellets obtained from Sigma‐Aldrich). These polymers were dissolved in dichloromethane (DCM, with a purity of ≥99.9% obtained from Sigma‐Aldrich) and sonicated for 10 min. Nafion (perfluorinated resin solution, with a concentration of 5 wt% in a mixture of lower aliphatic alcohols and water containing 45% water, obtained from Sigma‐Aldrich) was received in solution form and was dispersed in methanol. The MOFs, ZIF‐8 (Basolite Z1200 produced by BASF, Sigma‐Aldrich) and CALF‐20, were dried in a vacuum oven at 100 °C prior to use. The MOF was then added to the polymer solution, stirred for 30 min, and sonicated for another 30 min. The MMM solution was spray‐coated onto the backside of Ag/PTFE GDEs using a nitrogen spray gun (Paasche H‐3MH Single Action with a 0.65 mm head). The PGDEs were left to dry overnight and kept in vacuum conditions. The loading of MOFPL was determined by weighing the GDE substrate before and after spray coating, and a loading accuracy of 0.05 mg cm^−2^ was maintained in all the samples.

##### Electrochemical Performance Evaluation

The study on electrochemical CO_2_ reduction was conducted in two electrochemical cells. The control experiments and polymer screening studies utilized a custom‐made gas‐fed three‐electrode electrochemical cell with a surface area of 1 cm^2^. The cell had three compartments for feedstock gas diffusion, catholyte, and anolyte, separated by a gas diffusion electrode and a polymeric ion exchange membrane (AEM or BPM, sourced from fuelcellstore). Before testing, the AEM was kept in 1 M KOH for 24 h. An Ag/AgCl electrode (3.5 M KCl saturated with silver chloride, supplied by CH Instruments, Inc.) was used as the reference electrode, the eCO_2_R catalyst as the working electrode, and nickel foam (1.6 mm thickness, 350 g m^−2^ surface density, from MTI Corporation) as the counter electrode. The catholyte (1 M KOH or 1 M KHCO_3_) and anolyte (1 M KOH) were circulated at a flow rate of 40 mL min^−1^ using peristaltic pumps (from Cole‐palmer). The flow rate of the feedstock gas, which was a mixture of CO_2_ (99.999% purity, Air Liquide) and N_2_ (99.999% purity, Air Liquide), was controlled with a digital mass flow controller (Cole‐palmer, model: 32907‐63) and kept at 100 sccm. The experiments were conducted using a Bio‐Logic potentiostat (SP‐300) potentiostat/galvanostat. For the investigation of the effect of MOF material, optimization of the PGDE loading, and PGDE stability, a commercial membrane electrode assembly (MEA) electrolyzer (Dioxide Materials, with a surface area of 5 cm^2^) was used. The MEA was assembled with a nickel foam anode, 1 M KOH anolyte, and different membranes (BPM and AEM). The stability test was run at a constant current density of 30 mA cm^−2^ using a BPM membrane, nickel foam anode, and 1 M KOH anolyte. All electrochemical and product quantification experiments were repeated at least three times (*n* = 3) under identical conditions. Error bars represent ±1 to ±3 standard deviations from the mean. Gas product concentrations were determined using calibration curves (*R* ^2^ > 0.995), and variations across replicates were used to assess measurement uncertainty.

##### Reaction Product Analysis and Calculation

In all electrochemical experiments, the reactor outlet (effluent) flow rate of the gas stream was measured using a bubble flow meter prior to product quantification by gas chromatography. This ensured accurate quantification of gas products, calculation of FE and partial current density (*j*_*X*_), particularly under lean CO_2_ feed conditions where undesired CO_2_ loss and differing diffusivities of CO and H_2_ can significantly affect outlet volumes.

The quantification of the gaseous products of CO_2_ reduction, namely H_2_ and CO, was performed using a PerkinElmer Clarus 680 gas chromatograph equipped with Carboxen‐1000 and Molecular Sieve 5A packed columns. The instrument was equipped with a thermal conductivity detector and a flame ionization detector, and argon (with a purity of 99.999% from Air Liquide) was used as the carrier gas. The partial current density (*j*_*x*_) for product *x* can be calculated using the following equation:

(1) $$j_{x}=\frac{n_{x}{\overset{•}{v}}_{\text{gas}}c_{x}F}{V_{m}}$$

where *n*_*x*_ is the number of electrons transferred to produce 1 mole of product *x*. $\overset{•}{v}$_gas_ is the gas flow rate. *c*_*x*_ is the concentration of the product *x* detected by the gas chromatograph. *F* is the Faraday's constant (96 485 C mol^−1^). *V*_m_ is the unit molar volume at Standard Laboratory Conditions (SLC) (298.15 K and 100 kPa), i.e., 24.5 L mol^−1^.

The FE of gas product *x* (FE_*x*_) was determined employing the following equation:

(2) $${\text{FE}}_{x}=\frac{j_{x}}{j_{\text{total}}}\times 100$$

where *j*_total_ is the applied total current during the reaction and *j*_*x*_ is the partial current for product *x*.

In the experimental results presented in this article, the surface area of the electrode remained constant at 1 cm^2^, and all data regarding current densities and Faradaic efficiencies were obtained based on this fixed geometric area.

##### Scanning Electron Microscopy (SEM)

A field‐emission scanning electron microscope (FEI Quanta 400) with a 20 kV accelerating voltage was utilized to examine the morphology and surfaces of the sample. The elemental distribution was analyzed using an energy‐dispersive X‐ray spectroscopy (EDS) detector, and atomic mapping was carried out using TEAMTM EDS software.

##### Volumetric Measurements

CO_2_ and N_2_ adsorption isotherms were measured volumetrically on a Micromeritics 3Flex instrument at 25 °C. The sample (0.102 g) was loaded between two quartz filter disks in a flow‐through quartz tube (O.D. 12 mm). The samples were pretreated at 120 °C under vacuum for 12 h prior to each adsorption measurement.

##### XRD Analysis

The crystalline nature of the MOF materials and MOFs/PSF hybrids was determined by using XRD recorded on a Bruker D8 Discover instrument using Cu‐Kα radiation (40 kV, *λ* = 0.15418 nm) equipped with a LynxEYE 1D detector. The spectra were accumulated by using a scan size of 0.02° within a 2*θ* range of 10°–60°.

##### Raman Spectroscopy

Raman spectra were collected by a DXR3xi Rama imaging microscope equipped with a *λ* = 532 nm laser as the excitation source and a 50× objective lens. All spectra were recorded with a laser power of 2 mW over 150 acquisitions with an exposure time of ≈0.5 s.

##### Fourier‐Transform Infrared Spectroscopy (FTIR)

The Fourier‐transform infrared spectra of the PGDEs were collected using a Perkin‐Elmer Frontier FTIR spectrometer. The instrument was configured with a resolution of 4 cm^−1^, and the spectra were obtained through the accumulation of 32 scans per sample.

## Conflict of Interest

Patent Title: Permselective Gas Diffusion Electrode. Inventors: MG Kibria, GKH Shimizu, NN Marei, TA Al‐Attas, SK Nabil, US Patent App. 18/838,431, 2025.

## Supporting information

Supplementary Material

## Data Availability

The data that support the findings of this study are available in the supplementary material of this article.
