# Supplementary material for: Tailoring Polymeric Binder of Permselective Gas Diffusion Electrode for Low‐Concentration CO2 Electrolysis
Source: ChemSusChem. 2025 Oct 21;18(24):e202501145. doi: 10.1002/cssc.202501145 (PMC12703434; doi:10.1002/cssc.202501145)
Supplement: Supplementary file 1 — Supplementary Material [file CSSC-18-e202501145-s001.pdf]

Supporting information

Tailoring polymeric binder of permselective gas diffusion electrode for low-concentration CO_2 electrolysis

Hadi Shaker Shiran[1#], Shariful Kibria Nabil[1#], Tareq Al-Attas[1#], Karthick Kannimuthu[1], and Md Golam Kibria[1,*]

[1]Department of Chemical and Petroleum Engineering, University of Calgary, 2500 University Drive, NW, Calgary, Alberta T2N 1N4, Canada.

*Correspondence: md.kibria@ucalgary.ca

[#]These authors contributed equally to this work.

Supplementary Note 1

The mode of interaction of CALF-20 with PSF was analyzed using FTIR spectral studies (**Figure 5b)**. In PSF, the bands between 2800-3000 cm^{-1} denote C-H and C-O-C-CH_3 stretching, with aromatic C_6H_6 ring stretching at 1590 cm^{-1} [38]. Symmetric and asymmetric O=S=O group stretching appears at 1155 and 1295 cm^{-1},[39] while C-SO_2-C and C-O stretching are appeared at 1320 and 1015 cm^{-1}, respectively [40] Etheric C-O-C stretching is found at 1170 cm^{-1}, and vibrations below 1000 cm^{-1} imply carbon bending. Pristine CALF-20 comprises Zn centers with distorted trigonal bipyramidal geometry, where 1, 2-N atoms from 1, 2, and 4-triazolate are bridged to Zn. The N-4 extends to another Zn center with bidentate oxalates as secondary building units [26]. For CALF-20, the small bands near 3100 cm^{-1} correspond to a C-H vibration from a triazole ring, a band at 1560 cm^{-1} is from a C=N group, bidentate oxalate shows C=O at 1660 cm^{-1}, a ring strain of five-membered ring displayed an intense band between 1350-1550 cm^{-1}, and in-plane, out of plane bending vibrations showed accumulated bands at a low-frequency region. For the CALF-20/PSF 10 wt. %, most bands appeared from CALF-20 since the ratio is 9:1 and the low concentration of PSF makes it difficult to perceive the interactions. However, the appearance of low intense bands near 2800-3000 cm^{-1} from the ether linkage of PSF [40] and C-SO_2-C band also slightly deviated, indicating the interaction between the cationic Zn from CALF-20 and O from PSF **(Figure 5b)**. Raman spectral studies of CALF-20 and PSF membrane are shown in **Figure S8**. In PSF, the appearance of four main peaks at 790, 1140, 1580, and 3065 cm^{-1} are related to asymmetric C–S–C, asymmetric C–O–C, C_6H_6 ring, and C–H vibrations, respectively [41]. In CALF-20, a peak appeared at 1123 cm^{-1} due to the stretching of sp^2-C and N atoms of the triazole ring[26]. The Zn-N stretching and aliphatic C-H stretching peaks appeared at 292 cm^{-1} and 3150 $cm^{-1,}$ respectively. The azolate ring puckering and aliphatic C-H bending appeared at 680 and 1470 cm^{-1}. For the CALF-20/PSF, most of the CALF-20 peaks are retained. However, the reduced intensity at 790 and 1140 cm^{-1} indicates the interaction between the electrophilic Zn from CALF-20 with S and O of PSF [38]. Further, the aromatic ring peak and C-H peaks are diminished, indicating the hindrance effect of the CALF-20 moieties on the membrane. **Figure 5c** shows the model structure of CALF-20, and the magnified chemical structure of distorted trigonal bipyramidal Zn with a 1,2,4-triazole ring and sp2 hybridized carbon is portrayed in **Figure 5d**. Also, **Figure 5e** shows the CALF-20/PSF and the interaction with the electrophilic Zn. [42–44]. The thermal stability of the pristine CALF-20 and CALF-20/PSF 10 wt.% was studied by thermogravimetric analysis (TGA), as

shown in **Figure S9**. Three weight loss peaks appeared, one at 100 ºC for the evaporation of absorbed water, removal of the interacted functional groups at 350 ºC, and decomposition of carbon and ligands [45]. The lesser weight loss for CALF-20/PSF 10 wt.% compared to pristine CALF-20 at 350 ºC is because of the strong interaction with PSF.

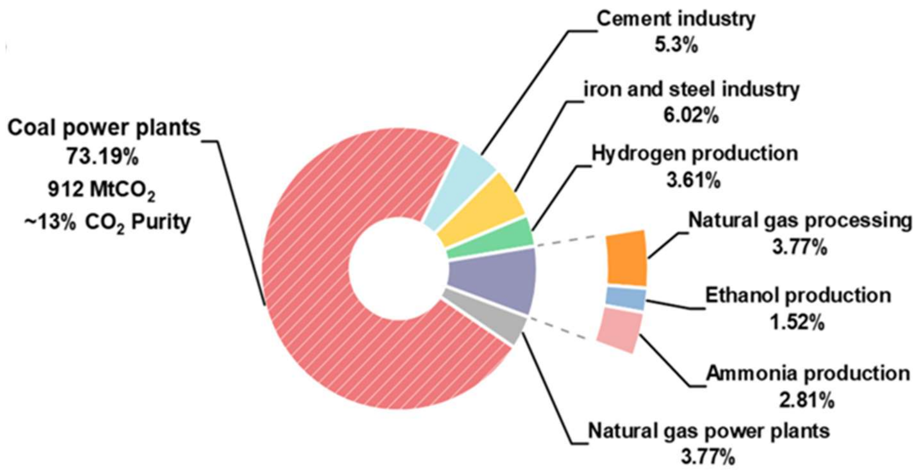

Figure S1. Distribution of annual CO_2 emissions from different point sources in the United States[5].

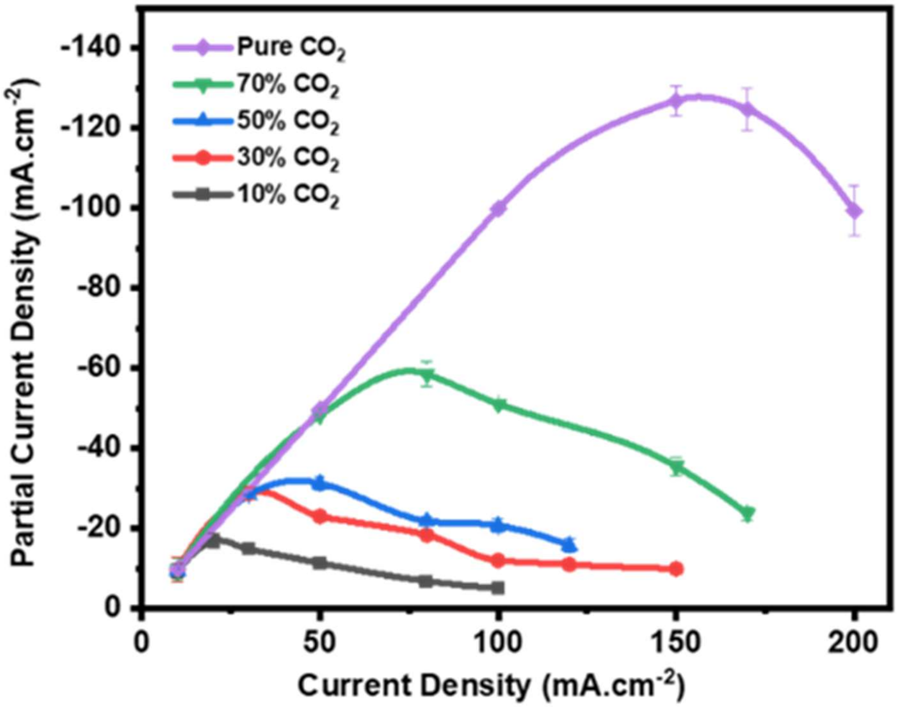

Figure S2. Partial current densities of Ag/PTFE at different lean CO_2 concentrations.

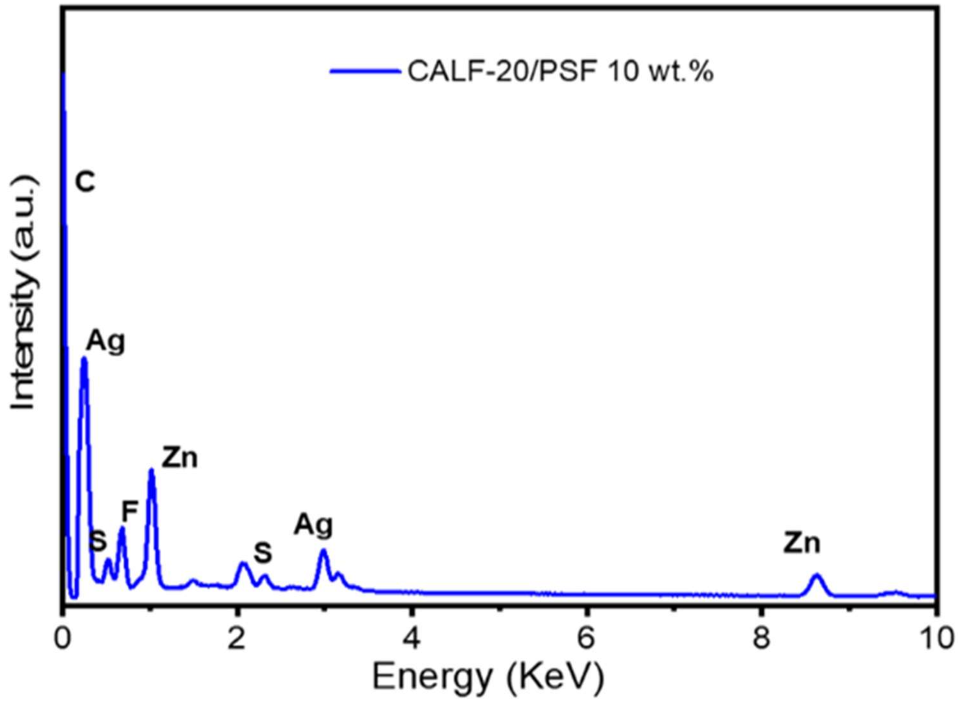

Figure S3. EDS analysis of CALF-20/PSF 10 wt% PGDE.

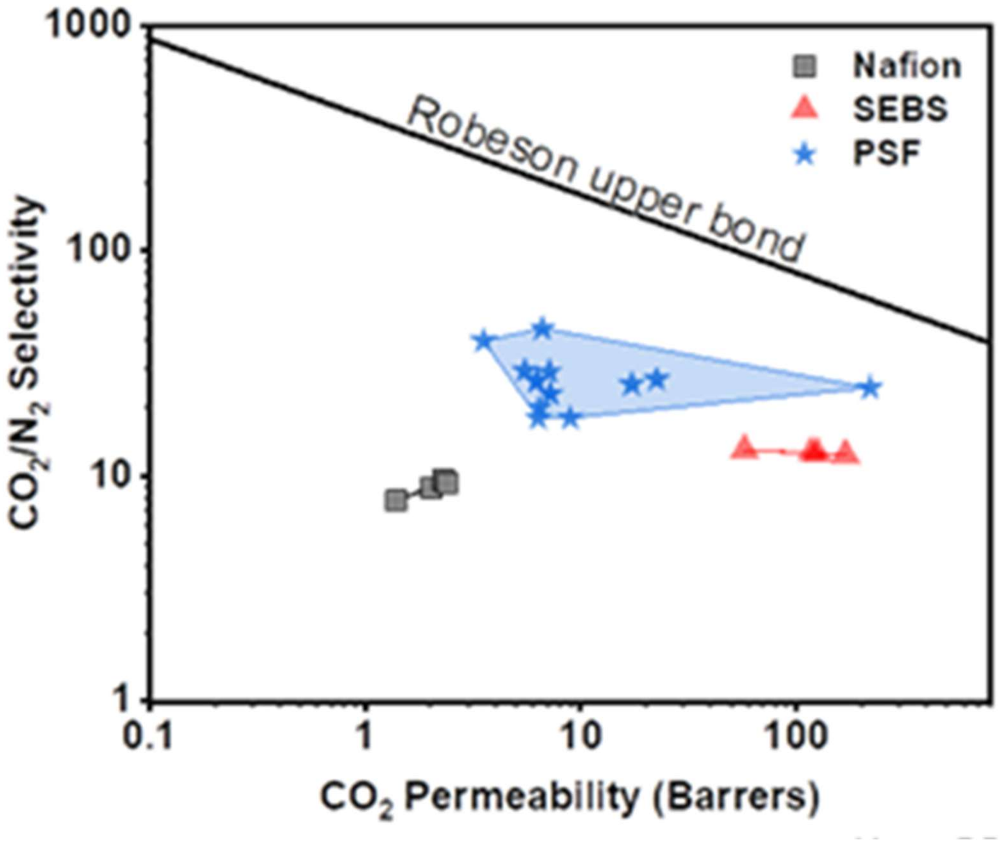

Figure S4. CO_2 permeability of pure polymeric membranes and their relation to Robeson upper bond (Details can be found in **Table S1 and S2**).

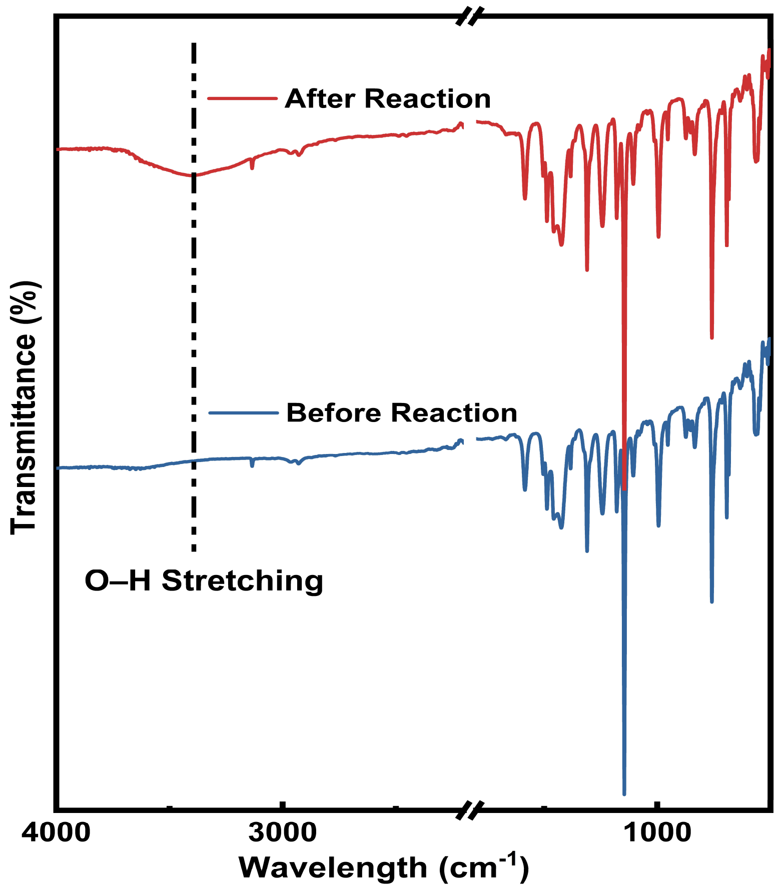

Figure S5. FTIR spectrum of the ZIF-8/PSF 10 wt.% before and after 2 h of eCO_2R.

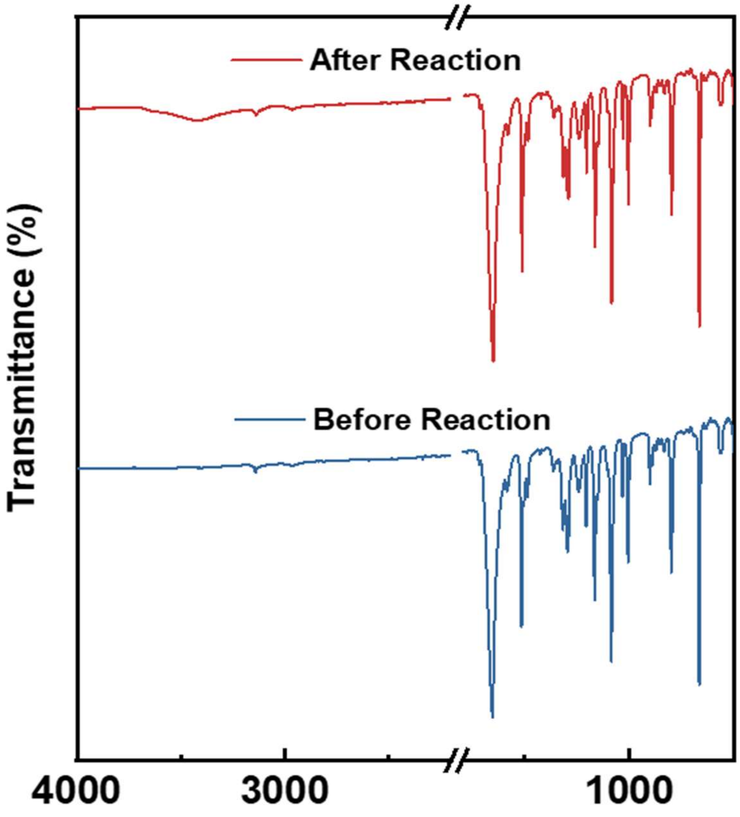

Figure S6. FTIR spectrum of the CALF-20/PSF 10 wt.% before and after 2 h of eCO_2R.

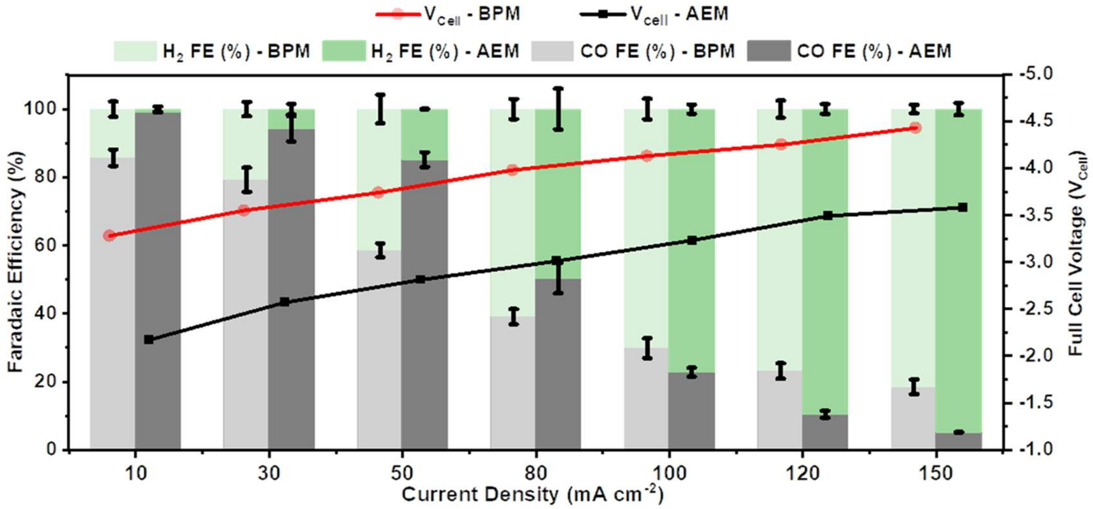

Figure S7. eCO_2R performance comparison of CALF-20/PSF using AEM and BPM in MEA using 1 M KOH anolyte,

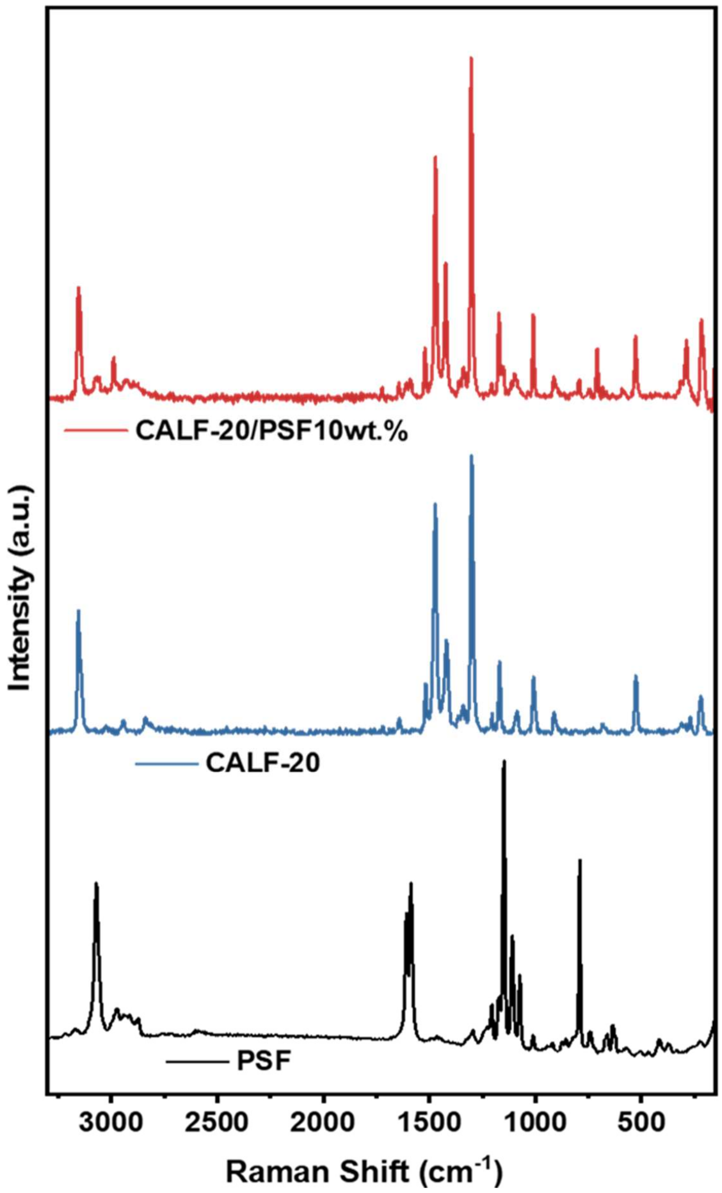

Figure S8. Raman spectra of CALF-20/PSF, CALF-20, and PSF.

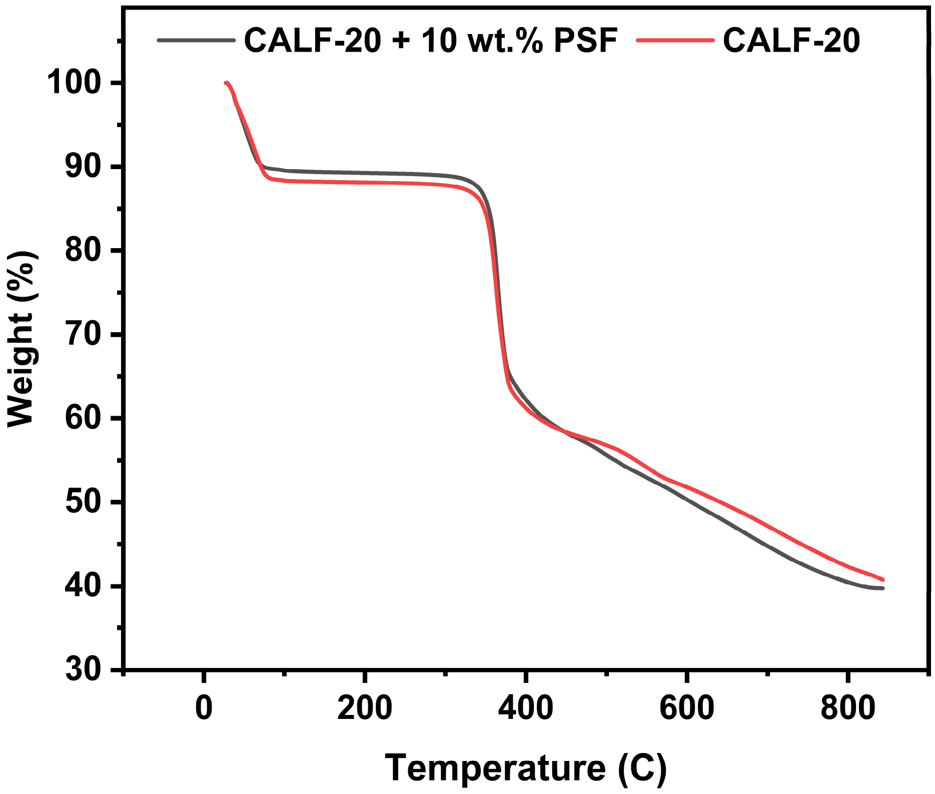

Figure S9. TGA analysis of CALF-20 and CALF-20/PSF 10 wt. %.

Supplementary Tables

Table S1. CO_2 permeability and CO_2/N_2 selectivities reported for pure polymeric membranes

Polymeric Membrane	Experimental Conditions	CO_2 Permeability (barrers)*	N_2 Permeability (barrers)*	CO_2/N_2 Selectivity	Reference
Nafion	Pure Gas - 2 atm and 35 °C	2.3	0.24	9.58	[2]
Nafion	Pure Gas - 1 atm and 35 °C	2.4	0.26	9.23	[2]
Nafion	Pure Gas - 4 atm and 30 °C	1.4	0.18	7.77	[2]
SEBS	Pure Gas – 0.5 MPa and 35 °C	170.6	13.8	12.4	[3]
SEBS	Pure Gas – 15 psi and 35 °C	126	10	12.6	[4]
SEBS	Pure Gas – constant volume/variable pressure method at 35 °C	58	4.3	13	[5]
PSF	Pure Gas - 1 bar and Room temperature	3.56	0.09	39.55	[6]
PSF	Pure Gas - 2 bar and 298K	6.32	0.24	26.33	[7]
PSF	Pure Gas - at 35 °C and Δp of 4.4 atm	6.3	0.24	26.25	[8]
PSF	Pure Gas - 5 bar	17.4	0.68	25.4	[9]

PSF	Pure Gas - 5 bar and room temperature	6.403	0.356	17.99	[10]
PSF	Pure Gas - 5 bar and 25 °C	65.24 GPU	3.88 GPU	17.26	[11]
PSF	-	~6.5	~0.325	~20	[12]
PSF	Wet condition - Pure Gas - 2 bar and 298K	6.29	0.24	26.21	[13]
	At 303 K and feed pressure of 6 bar	222	9.06	24.5	[14]
PSF	At room temperature and 1.5 bar	4.75	0.25	19	[15]
PSF	At room temperature and 2.5 bar	6.7	0.15	45	[15]
PSF	Room temperature	5.55	0.19	29.23	[16]
PSF	Ultrathin – 35°C	9	0.5	18	[17]
PSF	34°C and 4 atm	6.57	0.31	21.19	[18]
PSF	Room temperature and 10 bar	~6.5		28.9	[19]
PSF	Mixed gas – room T	7.2		28.8	[20]
PSF	Room temperature	7.3	0.3	23	[21]

* Permeability is reported in Barrers, where 1 Barrer = 10^{-10} cm^3(STP)·cm / (cm^2·s·cmHg)

Table S2. Robeson's upper bond constants and calculation

Gas Pair	Fitting Equation	n	K (barrers)	Reference
CO_2/N_2	$P_x = k^n$	-2.888	30.967×10^6	[22,23]

References

1. Lin, J. Bin *et al.* A scalable metal-organic framework as a durable physisorbent for carbon dioxide capture. *Science (80-.).* **374**, 1464–1469 (2021).

2. Mukaddam, M., Litwiller, E. & Pinnau, I. Gas Sorption, Diffusion, and Permeation in Nafion. *Macromolecules* **49**, 280–286 (2016).

3. Chi, W. S. *et al.* Mixed matrix membranes consisting of SEBS block copolymers and size-controlled ZIF-8 nanoparticles for CO_2 capture. *J. Memb. Sci.* **495**, 479–488 (2015).

4. Oh Lee, C. *et al.* SEBS-ionic liquid block-graft copolymer-based membranes with high compatibility for improved gas separation. *Eur. Polym. J.* **174**, 111309 (2022).

5. Tian, D. *et al.* Structure and gas transport characteristics of triethylene oxide-grafted polystyrene-b-poly(ethylene-co-butylene)-b-polystyrene. *J. Polym. Sci.* **58**, 2654–2663 (2020).

6. Miricioiu, M. G., Iacob, C., Nechifor, G. & Niculescu, V. C. High selective mixed membranes based on mesoporous MCM-41 and MCM-41-NH2 particles in a polysulfone matrix. *Front. Chem.* **7**, 332 (2019).

7. Sarfraz, M. & Ba-Shammakh, M. A novel zeolitic imidazolate framework based mixed-matrix membrane for efficient CO2 separation under wet conditions. *J. Taiwan Inst. Chem. Eng.* **65**, 427–436 (2016).

8. Ahn, J., Chung, W. J., Pinnau, I. & Guiver, M. D. Polysulfone/silica nanoparticle mixed-matrix membranes for gas separation. *J. Memb. Sci.* **314**, 123–133 (2008).

9. Surya Murali, R., Padaki, M., Matsuura, T., Abdullah, M. S. & Ismail, A. F. Polyaniline in

situ modified halloysite nanotubes incorporated asymmetric mixed matrix membrane for gas separation. (2014) doi:10.1016/j.seppur.2014.05.020.

10. Azizi, A., Feijani, E. A., Ghorbani, Z. & Tavasoli, A. Fabrication and characterization of highly efficient three component CuBTC/graphene oxide/PSF membrane for gas separation application. *Int. J. Hydrogen Energy* **46**, 2244–2254 (2021).

11. Zahri, K., Wong, K. C., Goh, P. S. & Ismail, A. F. Graphene oxide/polysulfone hollow fiber mixed matrix membranes for gas separation. *RSC Adv.* **6**, 89130–89139 (2016).

12. Car, A., Stropnik, C. & Peinemann, K. V. Hybrid membrane materials with different metal-organic frameworks (MOFs) for gas separation. *Desalination* **200**, 424–426 (2006).

13. Sarfraz, M. & Ba-Shammakh, M. Synergistic effect of incorporating ZIF-302 and graphene oxide to polysulfone to develop highly selective mixed-matrix membranes for carbon dioxide separation from wet post-combustion flue gases. *J. Ind. Eng. Chem.* **36**, 154–162 (2016).

14. Ban, Y. *et al.* Confinement of Ionic Liquids in Nanocages: Tailoring the Molecular Sieving Properties of ZIF-8 for Membrane-Based CO_2 Capture. *Angew. Chemie Int. Ed.* **54**, 15483–15487 (2015).

15. Singh, S., Varghese, A. M., Reddy, K. S. K., Romanos, G. E. & Karanikolos, G. N. Polysulfone Mixed-Matrix Membranes Comprising Poly(ethylene glycol)-Grafted Carbon Nanotubes: Mechanical Properties and CO_2 Separation Performance. *Ind. Eng. Chem. Res.* **60**, 11289–11308 (2021).

16. Waheed, N. *et al.* Mixed matrix membranes based on polysulfone and rice husk extracted

silica for CO_2 separation. *Sep. Purif. Technol.* **170**, 122–129 (2016).

17. Scholes, C. A., Chen, G. Q., Stevens, G. W. & Kentish, S. E. Plasticization of ultra-thin polysulfone membranes by carbon dioxide. *J. Memb. Sci.* **346**, 208–214 (2010).

18. Liu, Q. *et al.* Plasticization of a novel polysulfone based mixed matrix membrane with high-performance CO_2 separation studied by positron annihilation. *Colloids Surfaces A Physicochem. Eng. Asp.* **654**, 130108 (2022).

19. Ishaq, S., Tamime, R., Bilad, M. R. & Khan, A. L. Mixed matrix membranes comprising of polysulfone and microporous Bio-MOF-1: Preparation and gas separation properties. *Sep. Purif. Technol.* **210**, 442–451 (2019).

20. Saqib, S. *et al.* Sustainable mixed matrix membranes containing porphyrin and polysulfone polymer for acid gas separations. *J. Hazard. Mater.* **411**, 125155 (2021).

21. Dey, S. *et al.* Synthesis and Characterization of Covalent Triazine Framework CTF-1@Polysulfone Mixed Matrix Membranes and Their Gas Separation Studies. *Front. Chem.* **7**, 693 (2019).

22. Robeson, L. M. The upper bound revisited. *J. Memb. Sci.* **320**, 390–400 (2008).

23. Comesaña-Gándara, B. *et al.* Redefining the Robeson upper bounds for CO_2 /CH_4 and CO_2 /N_2 separations using a series of ultrapermeable benzotriptycene-based polymers of intrinsic microporosity. *Energy Environ. Sci.* **12**, 2733–2740 (2019).
